# Determinants of High Fasting Insulin and Insulin Resistance Among Overweight/Obese Adolescents

**DOI:** 10.1038/srep36270

**Published:** 2016-11-08

**Authors:** Jerri Chiu Yun Ling, Mohd Nahar Azmi Mohamed, Muhammad Yazid Jalaludin, Sanjay Rampal, Nur Lisa Zaharan, Zahurin Mohamed

**Affiliations:** 1 Sports Medicine, Deans’ Office, Faculty of Medicine, University of Malaya, 50603 Kuala Lumpur, Malaysia; 2 Sports Medicine Department, 11th Floor, South Tower, University Malaya Medical Centre, 59100 Kuala Lumpur, Malaysia; 3 Department of Paediatrics, University Malaya, 50603 Kuala Lumpur, Malaysia; 4 Department of Social and Preventative Medicine, Faculty of Medicine, University of Malaya, 50603 Kuala Lumpur, Malaysia; 5 The Pharmacogenomics Laboratory, Department of Pharmacology, Faculty of Medicine, University of Malaya, 50603 Kuala Lumpur, Malaysia

## Abstract

Hyperinsulinaemia is the earliest subclinical metabolic abnormality, which precedes insulin resistance in obese children. An investigation was conducted on the potential predictors of fasting insulin and insulin resistance among overweight/obese adolescents in a developing Asian country. A total of 173 overweight/obese (BMI > 85^th^ percentile) multi-ethnic Malaysian adolescents aged 13 were recruited from 23 randomly selected schools in this cross-sectional study. Waist circumference (WC), body fat percentage (BF%), physical fitness score (PFS), fasting glucose and fasting insulin were measured. Insulin resistance was calculated using homeostasis model assessment of insulin resistance (HOMA-IR). Adjusted stepwise multiple regression analysis was performed to predict fasting insulin and HOMA-IR. Covariates included pubertal stage, socioeconomic status, nutritional and physical activity scores. One-third of our adolescents were insulin resistant, with girls having significantly higher fasting insulin and HOMA-IR than boys. Gender, pubertal stage, BMI, WC and BF% had significant, positive moderate correlations with fasting insulin and HOMA-IR while PFS was inversely correlated (*p *< 0.05). Fasting insulin was primarily predicted by gender-girls (Beta = 0.305, *p *< 0.0001), higher BMI (Beta = −0.254, *p* = 0.02) and greater WC (Beta = 0.242, *p* = 0.03). This study demonstrated that gender, BMI and WC are simple predictors of fasting insulin and insulin resistance in overweight/obese adolescents.

An abnormally high insulin level or hyperinsulinaemia has been hypothesised as the earliest physiological risk marker of cardiovascular and metabolic diseases in obese children^1^. In obese children, hyperinsulinaemia can initially compensate well for insulin resistance^2^. However, chronic obesity may persistently impair insulin-mediated glucose metabolism^3^. Consequently, insulin resistance occurs as a response of the body to chronic hyperinsulinaemia^4^, resulting in glucose dysregulation^1^. The gold standard to measure insulin resistance is via the euglycaemic insulin clamp, which is a complicated and clinically impractical procedure as it requires three hours of continuous administration of both insulin and glucose intravenously^5^. Thus, in clinical practice, the evaluation of hyperinsulinaemia by measuring fasting plasma insulin may, therefore, provide a rational clinical alternative for evaluating insulin resistance^5^. Several models have incorporated fasting plasma insulin to indirectly measure insulin resistance, including a homeostasis model assessment of insulin resistance (HOMA-IR), a quantitative insulin sensitivity check index (QUICKI) and the fasting glucose/insulin ratio (FGIR). In children and adolescents, HOMA-IR has been shown to be more reliable than FGIR and QUICKI^6^ and has been used as a measure of insulin resistance in this population.

Similar to other countries worldwide, Malaysia, a multi-ethnic country in Southeast Asia is experiencing an increase in childhood obesity. In Malaysia, the 4^th^ National Health and Morbidity Survey 2011 reported a 6.1% prevalence of obesity among Malaysian children under 18 years of age, with higher rates occurring in urban areas^7^. A recent longitudinal cohort study involving 1,361 Malaysian adolescents had documented a 9% prevalence of obesity^8^, suggesting a continuous increase in the childhood obesity prevalence rate. These increasing trends are alarming, as childhood obesity is closely associated with increased risk of obesity and cardio-metabolic diseases in adulthood^9^. It is even more distressing that Malaysia had the second highest prevalence of diabetes mellitus in Asia, with a national diabetes prevalence of 10.9% in 2010^10^. It is postulated that the prevalence of type 2 diabetes mellitus in Malaysia will increase with the current trend of increasing prevalence of obesity in the adolescent population. Surveillance for fasting insulin and indirectly insulin resistance is thus beneficial, particularly among an apparently healthy overweight and obese paediatric population, in order to halt cardio-metabolic risk factors as they mature into adulthood.

Studies have reported that higher adiposity and lower cardiovascular fitness are significantly associated with higher fasting insulin in adolescents^11^,^12^. Often, dual-energy x-ray absorptiometry and maximal oxygen uptake are used as laboratory gold standard tests to quantify adiposity and cardiovascular fitness respectively^11^,^12^. However, these gold standard tests are expensive, time-consuming and clinically impractical for mass population surveillance, particularly in public health care and school settings. Previous studies involving paediatric populations had adopted several field tests as measurements of adiposity (i.e. waist circumference (WC), triceps and subscapular skinfold thickness, bioelectrical impedance analysis [BIA]) and cardiovascular fitness (i.e. step tests, physical working capacity at a heart rate of 170 [PWC_170_], 20m shuttle-run)^13^,^14^.

WC is an indicator of central obesity^15^ and therefore a good predictor of visceral fat^15^. Besides WC, waist to height ratio (WHtR), which incorporates WC as a measure of abdominal obesity and adjusts for an individual’s size by dividing by their height, may also be advantageous over BMI and waist to hip ratio in children^16^. BIA, as a measure of body fat percentage (BF%), is a validated tool and it is highly correlated with the gold standard dual-energy x-ray absorptiometry test^17^. Moreover, BIA is an easy, portable, and quick measurement tool that can be applied as a field test for mass screening. As for the cardiovascular fitness test, the step test has the advantage compared to other tests, as it is simple and not limited by availability of space or facilities, yet allows mass population testing to quantify aerobic fitness^18^.

Studies investigating the strength of low-cost but feasible mass population field tests to determine the predictors of high fasting insulin and consequently insulin resistance among adolescents are scarce, especially in countries in intermediate stages of nutrition transition such as Malaysia^19^,^20^,^21^. Hence, the aim of this study was to identify the predictors of high fasting insulin and insulin resistance (as measured by HOMA-IR) among overweight and obese Malaysian adolescents, by employing three clinically easily accessible instruments, namely a WC measuring tape, BIA, and the Modified Harvard Step Test (MHST)^22^,^23^,^24^ for mass population testing. In addition, this study also aimed to examine the relationship between fasting insulin and HOMA-IR with selected cardiovascular risk factors, namely blood pressure (BP) and lipid profiles among the adolescents.

## Results

A total of 173 overweight and obese adolescents completed all the study protocols whereby more than two-thirds were girls (n = 120), 80% were of Malay ethnicity with 7% Chinese and 8% Indians (Table 1). The majority of girls were already in the late pubertal stage (81%) in contrast to boys, more than half of the boys (55%) were still in the pre-pubertal stage. Maternal education as a measure of socioeconomic status^25^ revealed that most had secondary education (63%), less than a third had tertiary education. Nearly two-thirds of the adolescents (n = 108, 67% girls) were categorised as obese according to their BMI, whilst the remainder were categorised as overweight (n = 65, 73% girls) with an overall mean body mass index (BMI) ± Standard Error of 26.9 ± 0.3 kg/m^2^. In addition, girls had higher scores for a modified Child Nutrition Questionnaire (CNQ) which indicated healthier dietary patterns^26^ while reporting similar levels of physical activities using the Physical Activity Questionnaire for Children^27^.

The mean WC values for both boys and girls fell between the 90^th^ and 95^th^ percentile according to gender and age groups among Malaysian children (reference value: 83.8–93.3 cm for boys; 78.8–85.2 cm for girls). The mean WHtR values for boys also exceeded 0.5, the cut-off commonly used to define central obesity^28^. In addition, the overall mean fasting insulin was high at 20.7 ± 1.1 mU/L, which exceeded the 20 mU/L used as the cut-off to classify high fasting insulin or hyperinsulinaemia. HOMA-IR was also higher than the values which were used to classify insulin resistance (4.2 ± 0.2)^29^. The mean fasting insulin for girls was abnormally high (22.5 ± 1.5 mU/L) compared to boys who recorded borderline high fasting insulin (16.4 ± 1.4 mU/L) (*p *< 0.001). A third of the adolescents (33%) fulfilled the HOMA-IR criteria for insulin resistance with girls having significantly higher HOMA-IR levels than boys. The mean fasting blood glucose (FBG) level fell within the normal limit (<5.6 mmol/l)^30^ and was not significantly different between boys and girls statistically. Of note, only 2.3% (n = 4) of the adolescents had high FBG of more than 5.6 mmol/L with the maximum FBG recorded as 6.2 mmol/L among the adolescents. Compared to boys, girls demonstrated significantly higher mean BF% and resting heart rate, but significantly lower WC, WHtR and systolic BP. There was no significant difference in lipid profiles between boys and girls. Only 2% (n = 4) of these adolescents fulfilled the International Diabetes Federation (IDF) criteria for metabolic syndrome^30^. Results of the cardiorespiratory fitness test showed that boys performed better in the Modified Harvard Step Test, with a mean physical fitness score of 62.1 ± 1.1 (*p *< 0.001). The majority of these adolescents had either a low average physical fitness score (n = 80, 46%) or high average physical fitness score (n  =  74, 42%). The remaining 12% (n = 21) fell under the poor physical fitness score category. None achieved a good or excellent physical fitness score.

BMI had the highest, statistically significant positive correlation with fasting insulin (r = 0.51, *p *< 0.0001) and HOMA-IR (r = 0.48, p < 0.0001) (Table 2), indicating that higher BMI was associated with higher fasting insulin and insulin resistance. In addition, WC, WHtR and BF% demonstrated significantly positive moderate correlations with fasting insulin and HOMA-IR. As an indicator of cardiorespiratory fitness, physical fitness score demonstrated a weak negative correlation, indicating that a lower physical fitness score was associated with higher fasting insulin and insulin resistance. Interestingly, gender (female) and pubertal stage showed statistically significant positive correlation with fasting insulin and HOMA-IR. When high fasting insulin and insulin resistance were examined as categorical variables, BMI (adjusted OR = 1.48; 95% confidence interval (CI) 1.11–1.98, p-value 0.008) and WC (adjusted OR = 1.10; 95% confidence interval (CI) 1.01–1.20, p-value 0.04) showed significantly increased risk of hyperinsulinaemia in our overweight and obese adolescents. No significant relationship was observed between all variables and risk of insulin resistance (Supplementary Table 1).

The prediction model for fasting insulin was statistically significant (*p *< 0.001) and accounted for approximately 29% of the variance of fasting insulin among our population of overweight and obese adolescents (R^2^ = 0.29, adjusted R^2^ = 0.28). Fasting insulin was primarily predicted by (i) gender-girls (Standardized Beta Coefficients (Beta) = 0.305, *p *< 0.0001); (ii) higher BMI (Beta = −0.254, *p* = 0.02); and lastly (iii) a greater WC (Beta = 0.242, *p* = 0.03) (Table 3). BF%, WHtR, physical fitness score and pubertal stage were excluded as predictive variables by the stepwise multiple regression. The fitted model provided us with an equation: fasting insulin = −0.957 + 0.16 (girls) + 6.5 (BMI) + 0.006 (WC), where girls had 0.16 mU/L higher fasting insulin than boys. The model for HOMA-IR mirrored the model for fasting insulin (R^2^ = 0.27 and adjusted R^2^ = 0.26) and similarly predictors of HOMA-IR included BMI, gender and WC where HOMA-IR = −3.784 + 0.382 (girls) + 1.004 (BMI) + 0.014 (WC). Of note, the interaction effect between gender and other covariates (waist circumference and BMI) was not significant statistically. Paired scatterplots showing the relationship between the selected predictors and both fasting insulin and HOMA-IR are shown in Supplementary Figure 1.

Significant positive moderate correlation was found between fasting insulin and fasting triglycerides (r = 0.27, *p *< 0.0001) while HDL-cholesterol was negatively correlated to fasting insulin (r = −0.18, *p* = 0.003). For HOMA-IR, significantly positive moderate correlation was found for fasting triglycerides (r = 0.25, *p* = 0.001) with weak correlation found with systolic BP (r = 0.18, *p* = 0.02). Similarly, negative correlation was found between HOMA-IR and HDL-cholesterol (r = −0.18, *p *= 0.003). In regression analysis, only the triglyceride level was significantly associated with fasting insulin and HOMA-IR, when regression analysis was performed adjusting for confounding variables (Table 4).

## Discussion

The use of readily available measuring tools to assess overweight and obese Malaysian adolescents en masse had demonstrated that selected indices of adiposity (BMI, WC, WHtR and BF%) as well as gender and pubertal stage had significant positive correlations with fasting insulin and HOMA-IR. Secondly, physical fitness score, an indicator of cardiorespiratory fitness, was inversely correlated with fasting insulin and HOMA-IR. Thirdly, a fitted model consisting of gender, BMI and WC from stepwise multiple regression analysis explained more than a quarter of the variance of fasting insulin and HOMA-IR. In addition, this study also demonstrated that fasting insulin and HOMA-IR were significantly associated with triglyceride levels as one of the cardiovascular risk factors in our adolescent population.

BMI, though easily obtained, is in fact an indicator of overall adiposity^15^, and is incapable of differentiating between fat mass and fat-free mass^31^. Therefore, this study included the use of BIA to measure BF% and lean body mass in our overweight and obese adolescent population. However, our study had shown that compared to BF%, the use of the simple measure of BMI demonstrated higher correlation with fasting insulin and HOMA-IR. BMI was included in our stepwise multiple regression model to predict both fasting insulin and insulin resistance. BF% and lean fat mass were not included in the final model selection. Furthermore, our study is consistent with current available evidence that supports WC as a strong predictor for fasting insulin and insulin resistance among overweight and obese adolescents^32^,^33^. Bassali *et al*. reported that the WC percentile offers additional information regarding risk that is not detected by BMI among moderately obese children^32^. In their study involving seven to eleven-year-old obese children, they reported that WC had a sensitivity of 72%, specificity of 64%, and a positive predictive value of 87% for hyperinsulinaemia. Bassali *et al*. concluded that children with a larger WC were 3.7 times more likely to have high fasting insulin (fasting insulin > 90 pmol/L was used as the cut-off for abnormal level). In addition, by investigating 391 obese adolescents aged 10 ± 3 years, Bedogni *et al*. had shown that WC and BMI explained 13% and 9% of the variance of log-transformed fasting insulin respectively^21^, (which were much lower than those observed in our study). They concluded that WC was a stronger predictor of fasting insulin than BMI^21^. Although WHtR had shown to be moderately correlated with fasting insulin and HOMA-IR, it had failed to be included as a predictor in both our stepwise regression models.

Physical fitness score had a statistically significant weak negative correlation with fasting insulin in our adolescent population, reflecting that a higher fitness level was associated with favourable lower fasting insulin. Of note, there was considerable diversity in physical fitness score within the same level of fatness. For example, our data reported a diverse physical fitness score of 20.2 to 75.4 among obese girls (n = 74). This diversity may reflect the underlying reason for a weak correlation between physical fitness score and fasting insulin in our study. In adults, it has been postulated that a higher fitness score is associated with a substantial decrease in metabolic risk for a given level of fatness^34^. Previous studies by Eisenmann *et al*. also concluded that aerobic fitness attenuates the metabolic risk among fat children and adolescents, although different protocols were engaged as the measurement of aerobic fitness in each of these studies. For example, treadmill time to exhaustion, 1.6 km run, and physical working capacity at a heart rate of 170 (PWC_170_)^35^,^36^,^37^.

Other studies, using gold standard laboratory instruments (i.e. maximal oxygen uptake and dual-energy x-ray absorptiometry), to quantify fitness and fatness among children advocated both fitness and fatness as predictors of fasting insulin in adolescents^11^,^12^. Allen *et al*. concluded that fitness (as indicated by maximal oxygen uptake) is a stronger predictor of fasting insulin than fatness, which was represented by BF% obtained via dual-energy x-ray absorptiometry^11^. Suriano *et al*. conducted a study involving children aged six to thirteen years, with the Queen’s College Step Test as the assessment for fitness, WC and BMI as indicators of fatness. Their results indicated that fitness is a protective factor among healthy-weight children, and that a high fitness level is associated with a favourable metabolic profile, including lower fasting insulin^20^. The conflicting outcomes in our study may be in part explained by the differences in the application of assessment tools, age range and statistical analyses.

Our study demonstrated that by being overweight and obese, adolescent girls faced a higher risk of having a high fasting insulin and insulin resistance as compared to boys. To our knowledge, this gender difference had never been highlighted in other studies involving adolescents. A higher fasting insulin level among adolescent girls could be partly due to the influence of sex hormones, gender differences in fat distribution, or generally a more mature pubertal stage as compared to boys of the same chronological age. In our study, most girls already entered puberty whilst the majority of boys were still in the pre-puberty stage. In a recent longitudinal study with obese children, it was demonstrated that entering into puberty doubled the risk for them to change from being metabolically healthy obese (defined as absence of cardiovascular risk factors) to metabolically unhealthy obese children [OR = 1.9 (95% CI = 1.3–2.8), *p* = 0.004]. The risk tripled when growing from mid puberty to late puberty [OR = 3.1 (95% CI = 2.1–4.5), *p *= 0.001]. These are the areas yet to be explored and warrant future research to provide us with a better insight and understanding on how gender differences may contribute to fasting insulin level and insulin resistance in adolescents; and whether this will translate into differences in cardio-metabolic risk between the genders later in adulthood.

Attempting to measure morbidity and mortality endpoints in children can be challenging^20^ as children usually only manifest cardiovascular diseases much later in adulthood. Likewise, the prevalence of metabolic syndrome in our study was rather low (2%). Thus, cardiovascular risk factors rather than metabolic syndrome have been highlighted among the paediatric population^20^. Our study demonstrated that fasting insulin and HOME-IR was significantly associated with triglyceride levels, one of the criteria used in metabolic syndrome. Using fasting insulin alone yielded similar results as using HOMA-IR when relationships with cardiovascular risk factors were examined in our study. Previous studies have shown that hyperinsulinaemia is associated with cardiovascular risk factors^1^ and it can initially compensate well for insulin resistance in obese children^2^. Libman *et al*. reported a significantly positive relationship between both fasting and 2-hour insulin in relation to cardiovascular risk factors in overweight children, highlighting the importance of insulin surveillance in clinical practice^1^. The high prevalence of insulin resistance in our study population (33%) is worrying. The Bogalusa Heart Study which followed their paediatric cohort found that the prevalence of metabolic syndrome in adults was higher in the insulin-resistant obesity group than in the insulin-sensitive obesity group (34.9% vs. 24.3%, *p *= 0.008) and those with insulin-resistance were 1.7 times (*p *= 0.011) more likely to have metabolic syndrome in adulthood compared to those who were insulin-sensitive^38^.

In our study, although the schools were selected randomly across different zones in Kuala Lumpur, Malaysia, girls may have been overrepresented possibly due to volunteer bias. The interaction effect was tested between girls and other covariates and was found to be insignificant. Due to the small number of participants, separate analysis for boys and girls was not performed. Metabolic syndrome was not examined as only 2% (n = 4) fulfilled the criteria for metabolic syndrome in our study. Causality could not be determined due to the cross sectional design.

We acknowledged that performance in the step test is dependent on heart rate measurement^39^. It is likely that when performing under unfamiliar situations, participants may manifest a certain level of anxiety, resulting in a higher heart rate. In this cross sectional study, we were unable to assist the participants to practise and be familiarised with the step test beforehand. Prior practice or familiarisation of step testing will influence performance after habituation, which can lead to gradual reduction in the level of anxiety and thus simultaneously decreasing the heart rate^39^. However, our study setting mimics the environment of public health care, where time and space constraints can impose a substantial influence on the choice of tests. Thus step testing was advocated as the cardiorespiratory fitness test without any familiarisation in our study. We did not adopt more sophisticated tools such as accelerometers and pedometers as these instruments only provide measurements of physical activity. These tools would require adolescents to comply with a certain period of monitoring, that is seven consecutive days^40^ and non-compliance may thus be a potential drawback to data collection^40^, considering the study was done in schools. These instruments are also relatively expensive, time-consuming, and thus not feasible for mass testing. In addition, Galaviz *et al*. had investigated the associations between physical activity, cardiorespiratory fitness, and obesity in Mexican children^41^. They concluded that fitness, as measured by a 20m shuttle-run test, had a stronger correlation and was a better predictor of obesity than physical activity (measured by a pedometer) among the children^41^.

In conclusion, this study demonstrated that adiposity (BMI, WC, WHtR and BF%) and fitness (physical fitness score) correlate with fasting insulin and HOMA-IR, with gender, BMI and WC being significant predictors for high fasting insulin and insulin resistance. This study was done in a developing Southeast Asian country, hence extending previous findings that have been mostly reported from Western and developed countries. Primary prevention programs should focus on overweight and obese adolescents, particularly girls, in view of the significantly higher fasting insulin and insulin resistance in girls as documented. BMI and WC measurements are easily employed for clinical surveillance/screening of the overweight/obese paediatric population; particularly within the public health care system, where clinical practice is often subjected to financial, time and space constraints.

## Methods

### Participants & Study Protocol

This cross-sectional study recruited thirteen-year-old, apparently metabolically healthy overweight and obese adolescents. They were selected via multi-stage sampling from 23 randomly selected government-funded daily national secondary schools in Kuala Lumpur, Malaysia. In 2011, Cohort of Paediatric Obesity Working Research Group (CO-POWR) initiated a longitudinal study aiming to investigate the epidemiology of paediatric obesity and cardio-metabolic risks among adolescents in Kuala Lumpur, Malaysia. The study involved 1,113 adolescents (29% boys, 70% of Malay ethnicities). Ethics clearance was obtained from the Medical Ethics Committee of University of Malaya Medical Centre (UMMC) (Reference Number: MEC 896.123). Written approval to conduct the study was obtained from the Ministry of Education, Malaysia, the Federal Territory of Kuala Lumpur Education Department and the respective school principals. Written informed consent was obtained from all parents (or legal guardians) of participating adolescents prior to the study. Verbal informed consent was obtained from the participating adolescents during the study. This study was carried out in accordance with the International Conference on Harmonization – Guidelines for Good Clinical Practice (ICH-GCP) and the Declaration of Helsinki.

Of this CO-POWR cohort, 18% (n = 200, 33% boys) were categorised as overweight according to the International Obesity Task Force BMI criteria (BMI between the 85^th^ to 95^th^ percentiles for age and sex) and 10% (n = 112, 46% boys) were categorised as obese (BMI > 95^th^ percentile for age and sex). All overweight and obese adolescents (n = 312, 38% boys) were invited individually by invitation letters to join this study, with the recruitment period from 1^st^ May 2012 to 31^st^ October 2012. Adolescents with any medical illness, recent musculoskeletal injury or acute illness were excluded. Adolescents were provided with a briefing by the clinicians involved and were given an information sheet detailing the study.

### Demographic and Anthropometry Parameters

The data collection was conducted in the morning after an overnight fast of at least eight hours in the respective schools. Each adolescent disclosed self socio-demographic details such as gender, ethnicity, and date of birth by filling up a “Personal Data Sheet”. Parents or guardians of adolescents filled a demographic questionnaire including birth weight, breastfeeding and medical history of the adolescents. Demographic information of parents or guardians was collected too. Pubertal stage was self-assessed using coloured Tanner stages illustrations. Dietary patterns including food intake, health behaviour, attitude, environment and knowledge were assessed using a validated, adapted Malay version of the modified Child Nutrition Questionnaire (CNQ). Overall, a higher summary score indicated healthier dietary patterns^26^. Physical activity level (PAL) was self-reported with the 9-item Physical Activity Questionnaire for Children (PAQ-C); a summary score of 1 indicated low PAL while 5 indicated high PAL^27^.

A specific team of trained personnel performed the measurements of anthropometric parameters (height, weight, WC, BMI and WHtR), and BF% (Body Composition Analyzer Inbody 230, Biospace Co. Ltd, Korea), as detailed in Table 5.

### Clinical parameters

Systolic and diastolic BP and heart rate were measured as per Table 5. Trained clinicians collected a three-ml fasting whole blood sample from an antecubital vein of each adolecent using a Becton Dickinson Vacutainer Safety-Lok blood collection set. The samples were immediately labelled, stored in iceboxes and sent to the laboratory at the University of Malaya Medical Centre, Malaysia (laboratory accreditation: MS-ISO-15189) within 3 hours of collection. All samples were intact with no evidence of haemolysis. Each sample was centrifuged at 3500 revolutions per minute for ten minutes (Eppendorf Centrifuge 5810, Eppendorf, Germany) prior to measuring fasting insulin concentration via a chemiluminescent assay (Advia Centaur XP Immunoassay System, Siemens, USA). Serum triglycerides (TG), high-density lipoprotein (HDL) cholesterol and plasma glucose were analysed with a Dimension RxL Max (Siemens Healthcare Diagnostics Inc., Deerfield, IL). Low-density lipoprotein (LDL) cholesterol was calculated with the Friedewald equation^42^.

Insulin resistance was categorised based on HOMA-IR values proposed by Yin *et al*.^29^ whereby the cut-off used to define insulin resistance for prepubertal adolescents was HOMA-IR > 2.6; while for pubertal adolescents the cut-off was HOMA-IR > 3.2. Metabolic syndrome was defined using International Diabetes Federation (IDF) criteria^30^: WC ≥ 90^th^ percentile with ≥ 2 of the following: TG ≥ 1.7 mmol/L, HDL-C < 1.03 mmol/L, BP (≥130 mmHg systolic or ≥85 mmHg diastolic) or FBG ≥ 5.6 mmol/L.

### Cardiovascular Fitness Test: Modified Harvard Step Test

The Harvard Step Test was the earliest documented step test which was adopted during World War II for the selection of combat officers^43^. The Harvard Step Test protocol was initially performed with participants stepping up and down a 20-inch bench at a metronome beat of 120 beats per minute for five minutes, with the recording of the post-exercise heart rate to determine physical fitness. Over the years, the Harvard Step Test protocol has been modified to accommodate the high physical exertion level, as well as the demands on the lower limb muscles due to the high stepping height. The Modified Harvard Step Test protocols vary in terms of the step height (between 6 to 20 inches), stepping rates (between 72 to 144 beats per minute) and test duration (between two to six minutes). These step test protocols had been validated in healthy paediatric populations aged seven to eighteen years^18^,^44^,^45^.

The cardiorespiratory fitness of each adolescent was assessed with the MHST^22^,^23^,^24^, as described in Table 5. Adolescents sat and rested for five minutes in preparation for the step test. Their heart rate was constantly monitored with a finger pulse oximeter (NONIN GO_2_ Achieve 9570) that was attached to their right middle finger. Each adolescent was instructed to step up and down a thirty-centimetre high step box for five minutes, according to a 120 beat-per-minute metronome. Heart rate and oxygen saturation were initially recorded at 45 seconds, followed by another four readings taken at one-minute intervals. The MHST was terminated prematurely if our participants had a heart rate of higher than 200 beats per minute, oxygen saturation of less than 90%, shortness of breath, chest discomfort, or inability to complete the five-minute test for any reason. Upon completion or termination of the MHST, adolescents were instructed to sit down and rest. The total duration of the step test (in seconds) was recorded. The post-exercise heart rate was recorded at 0-minute, 1-minute, and 2-minutes. A physical fitness score was then calculated with the formula [duration of exercise (seconds)/sum of post exercise heart rate at 0-, 1 and 2-minutes] X 100^22^,^23^,^24^. The score was classified as poor (<55), low average (55–64), high average (65–79), good (80–89), excellent (≥90)^22^,^24^. The high average physical fitness score category (65–79) was the cut-off point to divide performance into the upper or lower physical fitness score tertile^22^,^24^.

### Data and Statistical Analysis

A total of 207 overweight and obese adolescents participate in the study. However, only 173 successfully completed all the study procedures. Statistical analysis was performed on the 173 adolescents (who had the complete sets of data) using Stata version 14.1 (Stata Corp, College Station, TX, USA), and *p* values of ≤0.05 were considered statistically significant. Weights were applied to samples to correct for unequal selection probabilities and non-responses because of multi-stage sampling. Complex sample (CS) univariate analysis was performed on weighted data to provide descriptive statistics (means and standard deviations). The parameters were tested for normal distribution using the Kolmogrov-Smirnov test and those that deviated from a normal distribution were log transformed for regression analysis. The variables that required log transformation include BMI, fasting insulin, HOMA-IR and physical fitness score.

Demographic characteristics between boys and girls were compared using Student’s t-test for normally distributed parameters and the Mann-Whitney U test for non-normally distributed parameters. The relationship between the variables of interest with fasting insulin and HOMA-IR were examined using Pearson’s correlation analysis for normally distributed variables and Spearman’s rank correlation analysis for non-normally distributed variables. In addition, the complex sample general linear model was used to determine the association between log-transformed fasting insulin, HOMA-IR and the cardiovascular risk factors (systolic and diastolic BP and lipid profiles) with analyses adjusted for the covariates mentioned.

Stepwise multiple regression analysis was conducted with continuous data for each variable to determine the predictors of fasting insulin, WC, BF% and physical fitness score. All models accounted for important confounders, age (in months), gender, BMI, ethnicity, puberty status, lean to fat mass ratio, maternal education as a measure of socio-economic status^25^, birth weight, history of exclusive breast feeding, dietary patterns using the CNQ scores, physical activity levels using PAQ-C scores, systolic and diastolic BP and fasting blood glucose. Logistic regression was used to examine the risk of hyperinsulinaemia and insulin resistance, with different adjustments for the confounders above. Goodness of fit of the model was examined using regression analysis and scatterplots. Assessment of leverage and outliership was performed using residual plots. Exclusion of outliers did not change the fit of model and thus all data were included in the final model.

## Additional Information

**How to cite this article**: Ling, J. C. Y. *et al*. Determinants of High Fasting Insulin and Insulin Resistance Among Overweight/Obese Adolescents. *Sci. Rep*. **6**, 36270; doi: 10.1038/srep36270 (2016).

**Publisher’s note:** Springer Nature remains neutral with regard to jurisdictional claims in published maps and institutional affiliations.

## Supplementary Material

Supplementary Information

## Figures and Tables

**Table 1:** Descriptive characteristics of participants in the study on determinants of fasting insulin and insulin resistance in overweight and obese Malaysian adolescents (n = 173 adolescents).

Characteristics	All (n = 173)	Boys (n = 53)	Girls (n = 120)	*p*-value
	n (%) or mean (±S.E)	n (%) or mean (±S.E)	n (%) or mean (±S.E)	
Age (years) *	12.9 ± 0.4	12.9 ± 0.4	12.9 ± 0.4	0.52
Ethnicity*				
Malay	138 (80)	45 (85)	93 (78)	0.30
Chinese	13 (7)	2 (4)	11 (9)	
Indian	14 (8)	4 (7)	10 (8)	
Others	8 (5)	2 (4)	6 (5)	
Pubertal stage* (n = 171)				<0.0001
Pre-puberty	31 (18)	28 (55)	3 (2)	
Early puberty	40 (23)	20 (39)	20 (17)	
Late puberty	100 (58)	3 (6)	97 (81)	
Birthweight (kg)	3.1 ± 0.7	3.4 ± 0.7	3.1 ± 0.6	0.03
Six-month exclusive breastfeeding*	30 (21)	8 (19)	22 (23)	0.59
Maternal education* (n = 118)				0.22
Primary	10 (8)	3 (8)	7 (9)	
Secondary	74 (63)	26 (72)	48 (61)	
Tertiary	34 (29)	7 (20)	27 (34)	
CNQ score*	3.0 ± 0.2	2.3 ± 0.3	3.3 ± 0.2	0.001
PAQ-C score	2.6 ± 0.1	2.7 ± 0.2	2.6 ± 0.1	0.68
Weight (kg)	64.7 ± 1.1	63.6 ± 1.6	65.2 ± 1.4	0.66
Height (cm)	154.6 ± 0.6	155.1 ± 1.3	154.4 ± 0.7	0.38
BMI* (kg/m^2^)	26.9 ± 0.6	26.4 ± 0.5	27.2 ± 0.4	0.16
WC (cm)	83.5 ± 0.9	85.6 ± 1.3	82.6 ± 1.2	0.01
WHtR	0.54 ± 0.01	0.55 ± 0.01	0.53 ± 0.01	0.02
BF%	41.5 ± 0.6	38.1 ± 7.7	42.9 ± 0.6	<0.0001
Lean fat mass (kg)	38.5 ± 1.4	39.62 ± 2.6	37.9 ± 1.7	0.80
Systolic BP (mmHg)	108 ± 1	112 ± 2	108 ± 1	0.01
Diastolic BP (mmHg)	65 ± 1	65 ± 2	66 ± 1	0.51
Resting HR (b.p.m.)	102 ± 1	97 ± 2	105 ± 2	0.02
PFS*	62.1 ± 1.1	66.0 ± 1.4	60.4 ± 1.4	<0.0001
Fasting blood glucose (mmol/L)	4.6 ± 0.1	4.6 ± 0.1	4.5 ± 0.1	0.29
Fasting insulin* (mU/L)	20.7 ± 1.1	16.4 ± 1.3	22.5 ± 1.5	<0.0001
HOMA-IR*	4.2 ± 0.2	3.4 ± 0.3	4.6 ± 0.3	<0.0001
Total cholesterol	4.24 ± 0.24	4.51 ± 0.16	4.56 ± 0.13	0.20
Triglycerides	1.07 ± 0.04	1.02 ± 0.07	1.10 ± 0.05	0.24
HDL-cholesterol*	1.25 ± 0.02	1.22 ± 0.04	1.26 ± 0.03	0.34
LDL-cholesterol*	2.78 ± 0.09	2.80 ± 0.13	2.78 ± 0.11	0.27

^*^Comparison of mean was examined using Man-Whitney U test, otherwise Student’s t-test.

BF%: body fat percentage; BMI: body mass index; BP: blood pressure; CNQ: Child Nutrition Questionnaire (CNQ); HOMA-IR: Homeostatic model assessment of insulin resistance; HR: heart rate; PAQ-C: Physical Activity Questionnaire for Children; PFS: physical fitness score; SE: standard error; WC: waist circumference; WHtR: waist-height ratio.

**Table 2:** Correlation between demographic, anthropometric and fitness factors with (a) fasting insulin and (b) HOMA-IR in overweight/obese Malaysian adolescents presented as correlation coefficient, R with *p*-values.

Characteristics	(a) Fasting Insulin		(b) HOMA-IR	
	R	*p*-value	R	*p*-value
Gender*	0.25	<0.0001	0.26	0.001
Age (years)*	−0.08	0.31	−0.08	0.31
Ethnicity *	0.01	0.87	−0.01	0.92
Pubertal stage*	0.21	0.01	0.20	0.01
Birthweight	−0.07	0.46	−0.07	0.47
Six-month Exclusive breastfeeding*	−0.10	0.26	−0.07	0.39
Maternal education	0.10	0.27	0.10	0.29
CNQ Score	0.10	0.20	−0.08	0.40
PAQ-C Score	−0.09	0.36	0.09	0.24
BMI*	0.51	<0.0001	0.48	<0.0001
WC	0.41	<0.0001	0.39	<0.0001
WHtR	0.35	<0.0001	0.34	<0.0001
BF%	0.34	<0.0001	0.32	<0.0001
Lean fat mass	0.30	0.07	0.28	0.09
PFS*	−0.19	0.01	−0.18	0.02

^*^Correlation was examined using Spearman’s correlation, otherwise Pearson’s correlation.

BF%: body fat percentage; BMI: body mass index; CNQ: Child Nutrition Questionnaire (CNQ); HOMA-IR: Homeostatic model assessment of insulin resistance; PAQ-C: Physical Activity Questionnaire for Children; PFS: physical fitness score; WC: waist circumference; WHtR: waist-height ratio.

**Table 3:** Prediction model of fasting insulin and HOMA-IR in overweight and obese Malaysian adolescents via adjusted stepwise multiple regression analysis.

Variables	Fasting insulin				HOMA-IR			
	b	*SE-b*	Beta	p-value	b	*SE-b*	Beta	p-value
Constant	−*0.957*	*0.449*	NA	NA	−3.784	*1.092*	NA	NA
BMI	0.442	0.189	0.254	0.02	1.004	0.460	0.240	0.02
Gender	0.16	0.038	0.305	<0.0001	0.382	0.091	0.303	<0.0001
WC	0.006	0.003	0.242	0.03	0.014	0.006	0.235	0.04

^*^Gender, pubertal stage, BMI, WC, WHtR, BF%, and PFS were included as variables in the adjusted stepwise multiple regression analysis.

*b*: unstandardized beta coefficients; Beta: Standardized Beta Coefficients; BF%: body fat percentage; BMI: body mass index; HOMA-IR: Homeostatic model assessment of insulin resistance; PFS: physical fitness score; WC: waist circumference; WHtR: waist-height ratio.

**Table 4:** Associations between cardiovascular risk factors and (a) fasting insulin and (b) HOMA-IR among overweight or obese Malaysian adolescents (n = 173).

Variables	Fasting Insulin*			HOMA-IR		
	Beta	R^2^	*p*-value	Beta	R^2^	*p*-value
Systolic BP (mmHg)	0.11	0.11	0.36	0.13	0.16	0.25
Diastolic BP (mmHg)	0.07	0.10	0.55	0.08	0.10	0.50
Total cholesterol (mmol/L)	0.22	0.10	0.07	0.22	0.10	0.07
Triglycerides (mmol/L)	0.35	0.13	0.004	0.32	0.12	0.009
HDL-cholesterol (mmol/L)	−0.08	0.18	0.52	−0.03	0.18	0.80
LDL-cholesterol (mmol/L)	0.17	0.06	0.18	0.16	0.05	0.20

^*^Adjusted for age, gender, ethnicity, pubertal stage, maternal education, BMI and WC.

Beta: Standardized Beta Coefficients; BMI: body mass index; BP: blood pressure; HDL: high-density lipoprotein; HOMA-IR: Homeostatic model assessment of insulin resistance; HR: heart rate; LDL: low-density lipoprotein; PFS: physical fitness score; WC: waist circumference.

**Table 5:** Measurement of blood pressure (BP), heart rate (HR), height, weight, waist circumference (WC), body mass index (BMI), Waist Height Ratio (WHtR), body fat percentage (BF%), and physical fitness score (PFS).

Measurement	Instrument	Remarks
BP and HR	• Omron HEM-97 Automatic Blood Pressure Monitor	• Participants sat in a chair with arm resting at the heart level
		• Measured with appropriate cuff size
Height	• Seca Portable 217 Stadiometer	• Bare foot
		• Measured to the nearest millimeter
Weight	• Seca Weighing Scale (Seca 813 Robusta High)	• Light clothes, bare foot
		• To the nearest decimal fraction of kg
WC	• Non-elastic Seca 201 Ergonomic Circumference Measuring Tape	• Landmark: Mid-point between the lowest margin of the last palpable rib and the top of the iliac crest (WHO STEPS protocol)
	• WC percentile values by age and sex^46^	• Measured to the nearest millimeter
BMI	• Calculator	• BMI = Weight (kg)/[Height (m)]
WHtR	• Calculator	• WHtR = WC (cm)/Height (cm)
BF%	• Body Composition Analyzer (Inbody 230)	• Bioelectrical Impedance Analysis
PFS	• Step box [45 (l) × 35 (w) × 30 (h) cm]	• Using Modified Harvard Step Test to measure cardiorespiratory fitness by estimating the ability of the cardiorespiratory system to adjust to and recover from hard work
	• Stopwatch	
	• Metronome 120 beats per minute	
	• Finger pulse oximeter (NONIN GO_2_ Achieve 9570) to monitor HR	
