# Supplementary material for: Determinants of High Fasting Insulin and Insulin Resistance Among Overweight/Obese Adolescents
Source: Sci Rep. 2016 Nov 8;6:36270. doi: 10.1038/srep36270 (PMC5099955; doi:10.1038/srep36270)
Supplement: Supplementary Information [file srep36270-s1.pdf]

Title

Determinants of high fasting insulin and insulin resistance among overweight / obese adolescents

Authors

Jerri Chiu Yun Ling[1, 2], Mohd Nahar Azmi Mohamed[1, 2], Muhammad Yazid Jalaludin[3], Sanjay Rampal[4], Nur Lisa Zaharan[5], Zahurin Mohamed[5].

Affiliations

[1]Sports Medicine, Deans' Office, Faculty of Medicine, University of Malaya, 50603 Kuala Lumpur, Malaysia. [2]Sports Medicine Department, 11th Floor, South Tower, University Malaya Medical Centre, 59100 Kuala Lumpur, Malaysia. [3]Department of Paediatrics, University Malaya, 50603 Kuala Lumpur, Malaysia. [4]Department of Social and Preventative Medicine, Faculty of Medicine, University of Malaya, 50603 Kuala Lumpur, Malaysia. [5]The Pharmacogenomics Laboratory, Department of Pharmacology, Faculty of Medicine, University of Malaya, 50603 Kuala Lumpur, Malaysia.

Supplementary Table 1: Risk of hyperinsulinaemia and insulin resistance in in overweight and obese Malaysian with selected predictors presented as odds ratio (OR) and 95% Confidence Interval (CI) with *p*-values.

Variables	Hyperinsulinaemia (Fasting insulin >20mU/L)		Insulin resistance (HOMA-IR >3)	
	OR* (95% CI)	*p*-value	OR*(95% CI)	*p*-value
Gender (Female)	6.04 (0.90, 40.49)	0.06	2.51 (0.46, 13.76)	0.29
Pubertal stage	3.13 (1.02, 9.64)	0.05	1.50 (0.59, 3.78)	0.39
BMI	1.48 (1.11, 1.98)	0.008	1.27 (0.94, 1.71)	0.12
WC (cm)	1.10 (1.01, 1.20)	0.04	1.02 (0.93, 1.12)	0.61
BF%	0.96 (0.87, 1.08)	0.47	1.04 (0.94, 1.15)	0.47
PFS	1.00 (0.95, 1.05)	0.99	1.01 (0.95, 1.07)	0.73

*Adjusted for age (months), ethnicity, maternal education, birth weight, breast-feeding status and WHtR

BF%: body fat percentage; BMI: body mass index; HOMA-IR: Homeostatic model assessment of insulin resistance; PFS: physical fitness score; WC: waist circumference; WHtR: waist-height ratio.

Supplementary Figure 1: Paired scatter plots showing the relationship between a) log-fasting insulin, and b) log-HOMA-IR and its predictors; waist circumference, gender and BMI.

a)

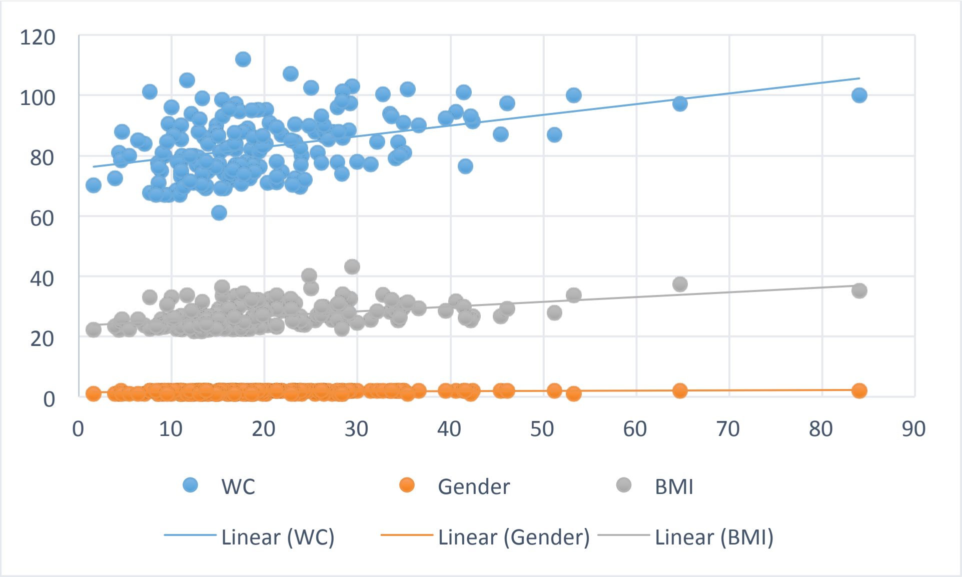

b)

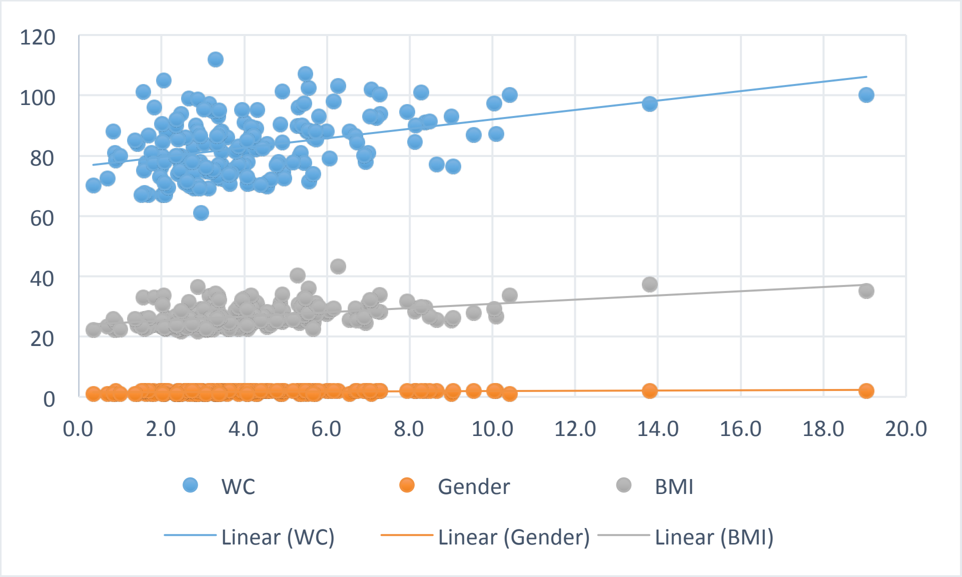

BMI: body mass index; HOMA-IR: Homeostatic model assessment of insulin resistance; WC: waist circumference.
